# Use and acceptance of long lasting insecticidal net screens for dengue prevention in Acapulco, Guerrero, Mexico

**DOI:** 10.1186/1471-2334-14-S2-P96

**Published:** 2014-05-23

**Authors:** Catrin Huws Jones, David Benítez-Valladares, Mario Barrera-Pérez, Felipe Dzul Manzanilla, Azael Che-Mendoza, Guillermo Guillermo-May, Johannes Sommerfeld, Axel Kroeger, Timothy O'Dempsey, Pablo Manrique-Saide

**Affiliations:** 1 Liverpool School of Tropical Medicine, Liverpool, UK; 2 University of Yucatán, Mérida, México; 3 Medical services of Yucatán, Yucatán, México; 4 Servicios Estatales de Salud de Guerrero, Chilpancingo, México; 5 Special Program for Research and Training in Tropical Diseases, México; 6 World Health Organization, Geneva, Switzerland

## Introduction

Dengue, recognized by the WHO as the most globally important mosquito-borne viral disease, is a growing problem. Currently, the only effective way of preventing dengue is vector control. Standard methods have failed, and there have been calls to develop new integrated vector management approaches. One novel tool, insecticide-treated window screens, is being trialed in a cluster randomized controlled trial by a joint UADY/WHO TDR study in Acapulco, Mexico, districts of which have exceptionally high levels of crime and insecurity. This study investigated the community's perspectives of insecticide-treated window screens, in homes and in schools, in order to ascertain their acceptability, identify challenges to further implementation and opportunities for future improvements.

## Methods

This was a sequential mixed-methods study. The quantitative arm contained two descriptive questionnaire surveys: a household survey administered to 2000 households prior to the intervention investigating dengue knowledge and attitudes, and a satisfaction survey administered to 288 houses that had received the intervention examining their perspectives of both the intervention and dengue prevention in general. The qualitative arm consisted of Focus Group Discussions (FGDs) with those who had accepted the intervention to discuss their perspectives in greater depth; and key informant interviews with: school teachers to discuss the use of the screens in schools, program staff to discuss their perspectives and experiences of implementing the project, and community members who had refused the intervention.

## Results

Overall satisfaction and acceptance of the screens was very high, with only some operational and technical complaints relating to screen fragility and the installation process. However, the wider social context of urban violence and insecurity was a major barrier to screen acceptance. Lack of information dissemination and community collaboration were identified as project weaknesses.

## Conclusion

The screens are widely accepted by the population, and could be a major new dengue prevention tool suitable for widespread use. The project implementation could be improved by reassuring the community of its legitimacy in the context of insecurity. More community engagement and better information sharing structures are needed. Further research is needed looking at the impact of insecurity on dengue prevention programs.

